# Isolation of the AP2/ERF transcription factor *CaERF14* in pepper and functional characterization under salinity and dehydration stress

**DOI:** 10.1038/s41598-025-03808-9

**Published:** 2025-06-05

**Authors:** Donglin Feng, Shuilin He, Jen-Ping Chung

**Affiliations:** 1 Fujian Vocational College of Agriculture, No.116 Guishan, Hongxing Village, Jingyang Town, Fuqing City, Fuzhou 350119, Fujian China; 2 Fujian Agriculture and Forest University, No.15 Shangxiadian Road, Cangshan District, Fuzhou 350002, Fujian China

**Keywords:** Pepper, Ethylene-responsive factor, Functional expression, Salt stress, Drought stress, Transgenic plant, Biotechnology, Molecular biology, Physiology, Plant sciences

## Abstract

Ethylene-responsive factors (ERF) are typically considered to play diverse roles in the plant’s response to biotic and abiotic stresses. In this study, an ERF gene, *CaERF14* was isolated from the pepper transcriptome database. The gene is 1,572 bp in length, containing an open reading frame (ORF) of 849 bp, which encodes a putative polypeptide of 283 amino acids with a theoretical molecular weight of 31.75 kDa. At the N-terminal, there is a conserved sequence of CMX-1 specific to the Xa subfamily of the ERF family. *CaERF14* is located in the nucleus and part of the cell membrane, interacting with the GCC-box to regulate the expression of downstream related genes. Results from the expression profile show that *CaERF14* has a higher expression level in parts of the floral and stem. Additionally, this gene can not only respond to the drought, NaCl, and *Ralstonia solanacearum*, but also be induced by signaling molecules (ABA, MeJA, Ethephon, SA, and auxins). Furthermore, the *CaERF14* derived from peppers was transformed into the model plant tobacco. Regardless of the NaCl, mannitol, or soil drought stress tested for seed germination or seedling growth in in vitro or in vivo culture, this gene exhibits salt and drought tolerance function. These results suggest that *CaERF14* is involved in the regulation of defense response to salinity and dehydration stress.

**Supplementary Information:**

The online version contains supplementary material available at 10.1038/s41598-025-03808-9.

## Introduction

APETALA2/ETHYLENE-RESPONSIVE FACTOR (AP2/ERF) transcription factors mainly exist in plants and were first reported in the *Arabidopsis* AP2 mutant^1^. AP2/ERF gene is defined by an AP2 domain containing 60–70 conserved amino acid residues, forming many transcription factors (including 147 genes in *Arabidopsis*). This family plays an important regulatory role in many biological and physiological processes, such as plant morphogenesis, response mechanisms to various stresses, hormone signal transduction, and metabolite regulation^2,3^. Based on the number of AP2 and other DNA binding domains, the AP2/ERF family could be divided into four major subfamilies and others, including AP2 (APETALA2), RAV (related to ABI3/VP), ERF (ethylene responsive element binding protein), DREB (dehydration responsive element binding), and a few unclassified factors-Soloist^4^. AP2 subfamily members play an important regulation role in plant growth and development, such as leaf epidermal cell identity and the development of flowers and ovules. Members of the RAV subfamily are involved in regulating plant development and response to various stresses. Meanwhile, ERF and DREB subfamily members are mainly involved in responding to biotic and abiotic stresses^3–6^. According to similarity and the number of DNA-binding domains, the family is diversely involved in the regulation of plant growth, development, metabolism, and stress response^7,8^.

ERFs were initially isolated from tobacco. Those transcription factors regulate biotic and abiotic stresses by binding to the GCC-box or dehydration responsive element (DRE)/C-repeat element (CRT), such as *ORA59* and *ERF1* gene in *Arabidopsis*, *Tsi1* in tobacco and *Pti4* in tomato^9–14^. Furthermore, some ERFs were also identified in pepper. *CaERFLP1* could form a specific complex with both the GCC box and DRE/CRT motif and respond to *Pseudomonas syringae* infection and salt stress in transgenic tobacco plants^15^. *CaCBF1 A*, *CaCBF1B*, *CaPF1* and *CaDREBLP1* could respond to different stresses, such as low-temperature, dehydration, high salinity, and wounding^16–18^. *CaRAV1* was demonstrated to be a transcriptional activator in triggering resistance to *Xanthomonas campestris* cv. vesicatoria. infection. Virus-induced gene silencing of *CaRAV1* and *CaRAV1*/*CAOXR1* confers enhanced susceptibility to high salinity and osmotic stresses^19,20^. *CaAP2* was also considered a candidate gene to control the flowering time in pepper^21^. ERF family has been identified and analyzed in horticulture crops, such as grape, tomato, cucumber, potato, pineapple, and strawberry^22–27^. Under stress conditions, protein members positively or negatively regulate defense responses, leading to plant adaptation. These protein family members play important modulatory roles in response to ABA-mediated dehydration signaling and are crucial for lateral root development^28,29^. Lateral roots in model plant *A. thaliana* originate from preselected pericyclic cells, which divide to form lateral root primordia, eventually growing to form a new root meristem. This process provides a mechanism of resistance to environmental stresses, particularly soil drought and salts.

Pepper (*Capsicum annuum* L.) is a significant and widely cultivated vegetable crop globally. In the cases of salt stress, drought stress and *R. solanacearum* resistance, specific genetic pathways of tolerance have been addressed^30–34^. Recent studies have shown that a pepper ERF family member, named *CaPTI1*, has the highest expression level in roots and responds not only to *Phytophthora capsici* infection but also to cold and drought stresses^35^. Drought stress induces a series of injuries in terms of plant physiological, biochemical, and metabolic impacts, resulting in plant growth retardation, cell damage, and loss of crop yield and quality^36,37^. Hong et al. (2017) reported that *CaAIEF1* positively regulates the drought stress response and ABA signaling in pepper^7^. The pepper AP2 domain-containing transcription factor *CaDRAT1*, belonging to the ERF subfamily, is significantly induced after exposure to abscisic acid (ABA), mannitol, low temperature, and H_2_O_2_^7^. Yang et al. (2021) studied the *ERF2* gene, significantly upregulated in both resistant and susceptible tomato cultivars in response to *Stemphylium lycopersici*^38^. *ERF2* plays a key role in salicylic acid (SA) and jasmonic acid (JA) signaling pathways, conferring resistance to invasion by *S. lycopersici*. Lee et al. (2020) demonstrated that JA synthesis, JA signaling, and ERF family genes contribute to the chilling response in pepper fruit^39^. This study can help elucidate the cellular mechanism or identify key factors affecting the chilling sensitivity or insensitivity of peppers following harvest.

In this study, we isolated and functionally characterized a member of the ERF subfamily B-4, the *CaERF14* gene. *CaERF14* affected dehydration resistance through the modulation of ABA biosynthesis and ABA sensitivity expressions of defense response-related genes, contributing to the resistance of tobacco to high salt and drought. Our findings imply that *CaERF14* functions as a regulator of plant dehydration and salinity stress response. In the process of global climate change, this study of the AP2/ERF factor provides important bases for understanding plant regulatory mechanisms in molecular breeding.

## Results

### Cloning and sequence analysis of *CaERF14*

**Fig. 1:**
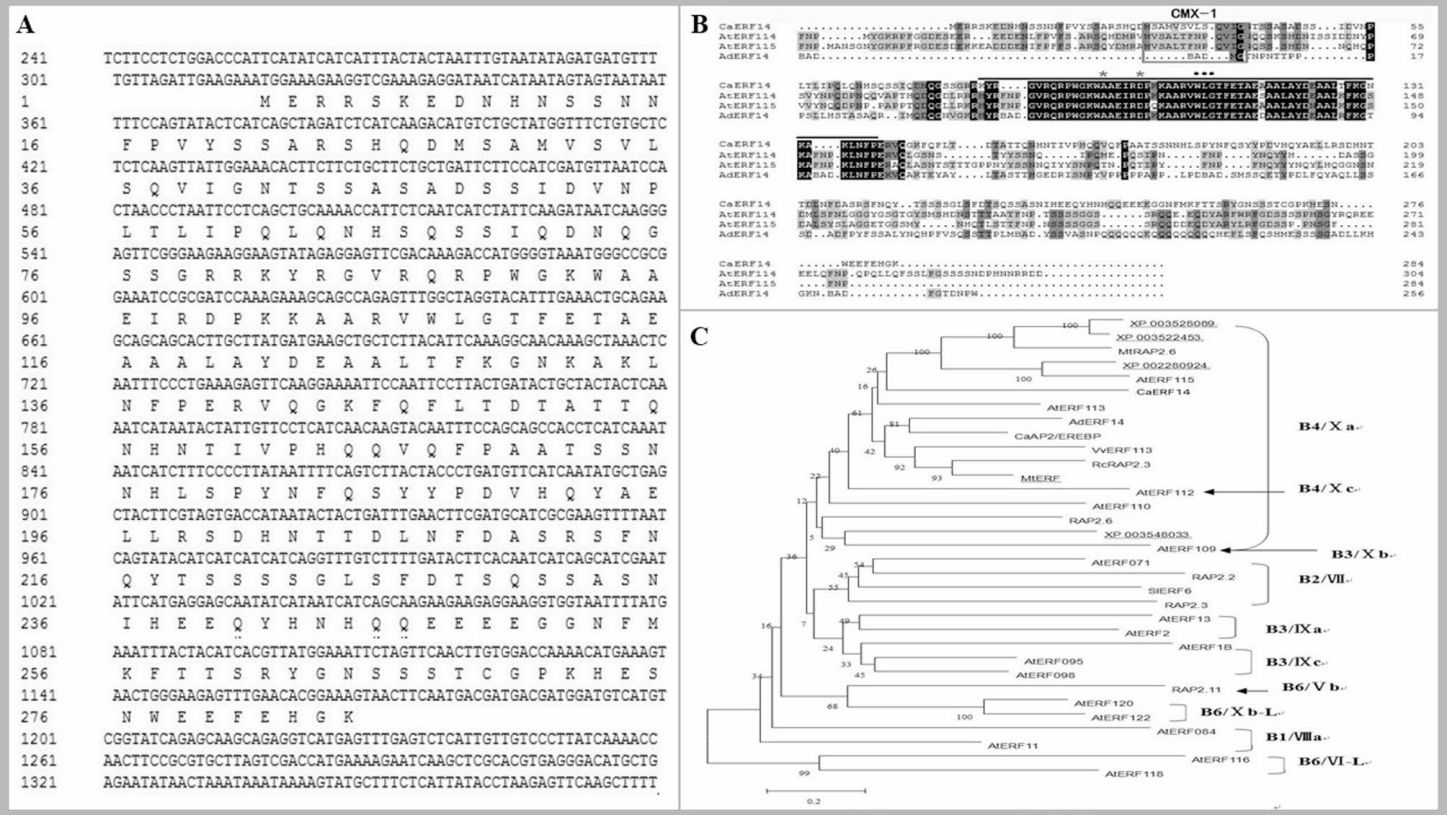
A new ERF gene *CaERF14* was isolated from pepper. **(A)** cDNA sequence and deduced amino acid sequence. **(B)** Multiple sequence alignment analysis of AP2/ERF domain containing *CaERF14* transcription factor with other plant homologous proteins. The number on the right represents the sequence position of amino acid residue. The upper line reveals the conserved AP2/ERF domain, and the asterisks indicate the A14 and D19 ERF specific amino acid residue. The black dots represent the conserved WLG motif. The gray boxed sequence represents the Xa subgroup specific motif. *AtERF114* and *AtERF115* are the AP2 transcription factors of *Arabidopsis*. *AdERF14* is the AP2 transcription factor of kiwifruit. **(C)** Phylogenetic tree shows the relationships of *CaERF14* with other species. The accession number indicates unnamed genes.

Molecular alignment of expressed sequence tags (ESTs) showed that the full-length *CaERF14* gene was screened and cloned from pepper cDNA library (Fig. 1A). Domain in comparison with the NCBI website, and the sequence analysis to identify putative the AP2/ERF domain-containing gene were analyzed by DNAMAN software. The *CaERF14* gene was 1,572 bp in length, which included an open reading frame (ORF) of 849 bp, and was identified from pepper transcriptome database. The ORF of *CaERF14* was predicted to encode a protein of 283 amino acids, with a predicted molecular weight of 31.75 kDa. According to the comparison results on the BLAST website, this gene has 70% homology with *Arabidopsis AtERF114* (NP_200995.1), 59% homology with *Arabidopsis* transcription factor *AtERF115* (NP_196348.1), and 57% homology with kiwifruit *AdERF14* (adj67443.1) (Fig. 1B; Supplementary Figure S2).

The expression patterns of the *CaERF14* revealed the presence of a conserved AP2/ERF DNA binding domain. Amino acids differences at position 14 th and 19 th in the AP2/ERF domain were also observed in these ERFs (Fig. 1B). The ERF subfamily differed from the unique alanine and aspartic acid of DREB subfamily and contained a landmark conserved WLG motif. At the N-terminal, there was a conserved sequence of CMX-1 specific to the Xa subfamily of the ERF family (Fig. 1B).

Furthermore, the evolutionary relationships and multiple sequence alignments of *CaERF14* in comparison with ERF proteins of other plants were also investigated (Fig. 1C). The results indicate that the newly obtained *CaERF14* gene can be confirmed as the Xa (B-4) subfamily of the ERF family (Fig. 1A-C).

### Tissue-specific expression of *CaERF14* gene in pepper plants

**Fig. 2:**
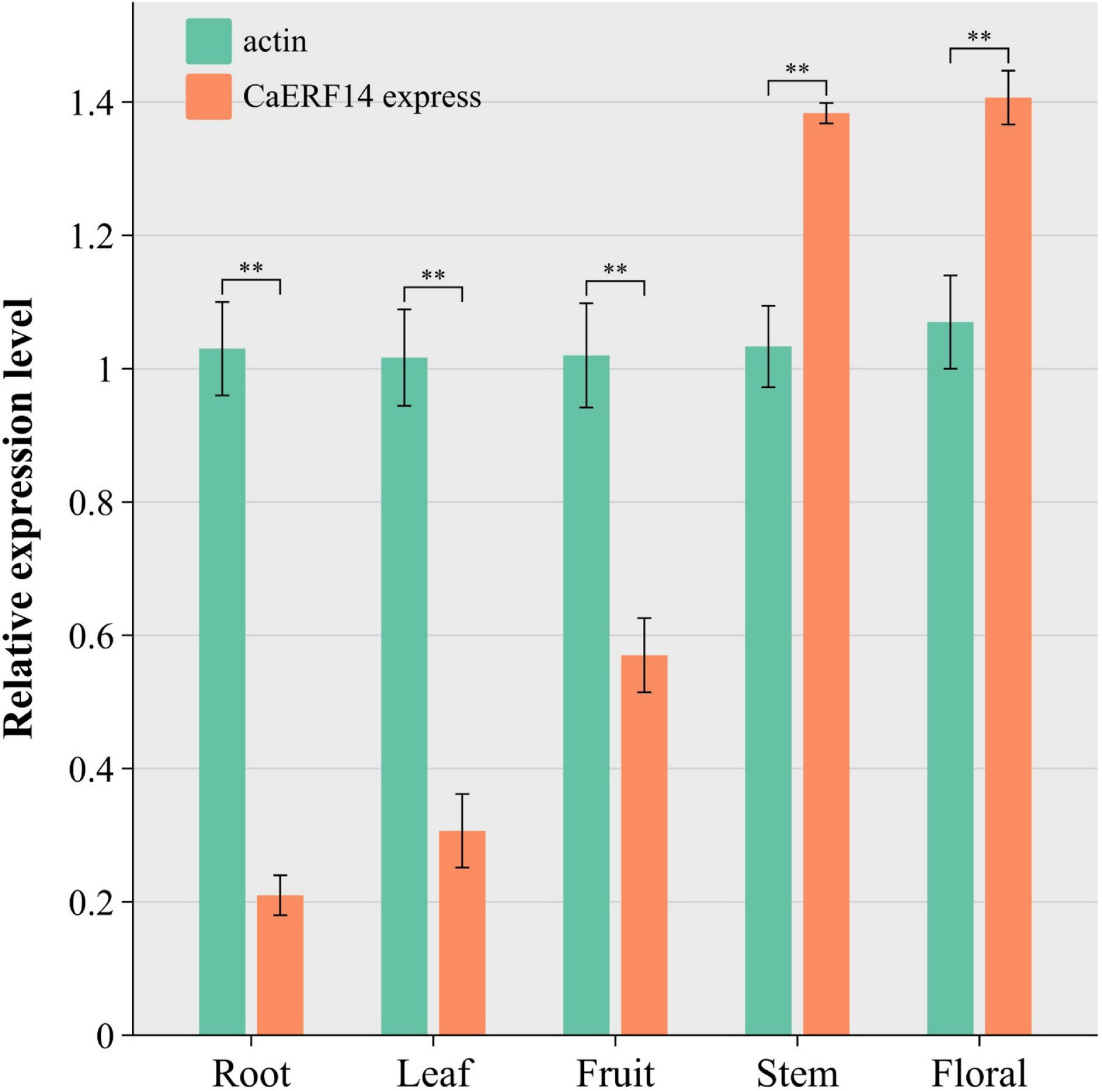
Quantitative real-time PCR expression level of *CaERF*14 in pepper different tissues. The actin gene was used as an internal control. R: Roots; S: Stems; L: Leaves; Fl: Flowers; Fr: Fruits. The vertical bar shows mean ± SD (*n* = 3). Asterisks indicate statistical significance (*0.01 < *P* < 0.05, **P < 0.01, Student’s t-test).

The expression levels of the *CaERF14* gene in different tissues of pepper plants were investigated by the MIQE guidelines: quantitative real-time PCR (qRT-PCR)^40^. A significant difference in *CaERF14* gene expression was observed among different tissues (Fig. 2). The highest expression was detected in floral parts, nearly 7-fold of that of roots. The expression level of *CaERF14* gene in stems was slightly lower than that in flowers, approximately 6.5-fold of that of roots. Lower expression was detected in fruits, leaves and roots as compared to the floral and stem parts. Particularly, the *CaERF14* gene showed the lowest expression in the roots. The tissue-specific expressions of *CaERF14 gene* in pepper were significantly different from the background actin gene, indicating that the gene expression results were feasible. In particular, the characteristics of *CaERF14* gene showed high expression in aboveground tissues, but lowest expression in underground parts such as roots.

### Transcriptional alteration of *CaERF14* in response to plant growth regulators

**Fig. 3:**
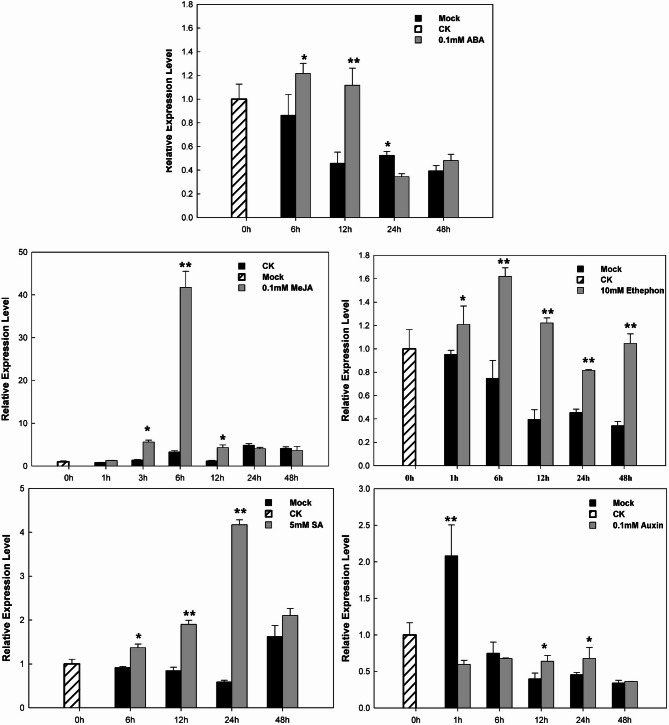
Relative expression of *CaERF14* responded to various plant growth regulator treatments as determined by quantitative RT-PCR. Relative expression of *CaERF14* gene in pepper seedlings following different treatments [abscisic acid (ABA), methyl jasmonate (MeJA), Ethephon (ETH), salicylic acid (SA) and auxin]. Mock represented plant with the mock treated as the control. Error bars represent the mean ± SD of three independent biological replicates. Asterisks indicate statistical significance *0.01 < *P* < 0.05, **P < 0.01, Student’s t-test.

The treatment of plant growth regulator is an important basis for confirming ERF family. In this study, the signaling molecules (ABA, MeJA, ethylene, SA, and auxin) were used to investigate the *CaERF14* gene (Fig. 3). The relative expression of *CaERF14* in pepper seedlings was significantly induced by foliar spraying of ABA, MeJA, Ethephon (ETH), and SA. Ethylene plays a crucial role in plant stress response, including the response to pathogens, and *ERF* gene expression. The qRT-PCR analyses were performed to investigate the expression in pepper seedlings, which was significantly induced by Ethephon treatment. In the changes from 0 to 24 h after treatment, the highest expression of *CaERF14* was detected at 6 h, and then gradually decreased to the lowest at 24 h, and returned to the background value (0 h) at 48 h. In comparison between the treatment group and the control (Mock) at each time point, the expression level of the treatment group at the 12-h point was more than 3-fold of the control, while the comparison test statistics of the two groups after 6 h of treatment showed extremely significant (*P* < 0.01, Student’s t-test). It showed that the *CaERF14* was sensitive to ethylene.

In the treatments with MeJA and SA, both the highest expression of *CaERF14* was detected at 6 h and 24 h, showing 12.6-fold and 9.2-fold of the control respectively. While in the treatment with ABA, the significant expression of induction was observed at 6 and 12 h post treatments, which was 1.4-fold and 2.5-fold of the control respectively. In addition, in the 0.1 mM auxin treatment, statistical analysis showed that there was a significant difference between the treatment group and the control (Mock) at 12 and 24 h post-treatment. However, the 1-h point showed that the expression level of the control (Mock) was greater than 2 times the background value (0 h), which may be a test error, but did not affect the auxin treatment effect.

### Expression analysis of *CaERF14* gene under abiotic and biotic stresses

**Fig. 4:**
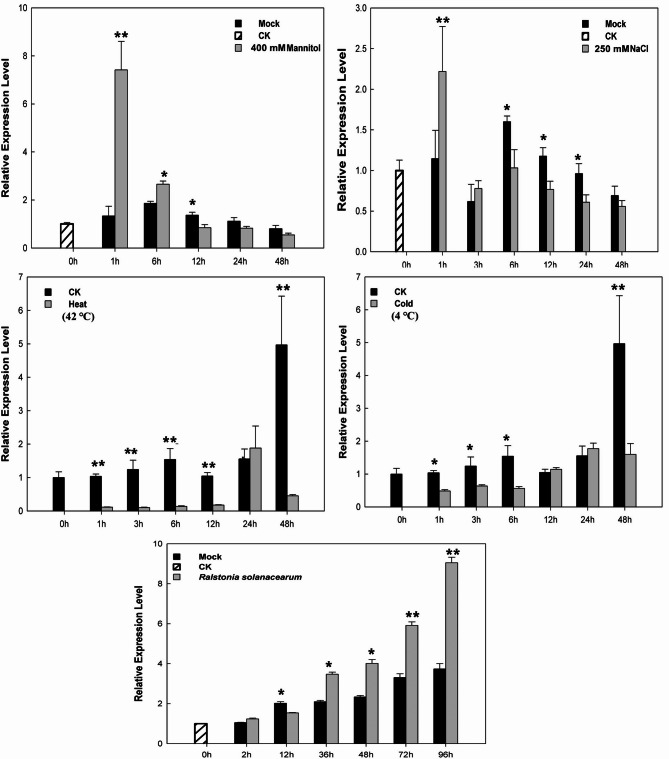
Relative *CaERF14* expression in pepper by different biotic and abiotic treatments as determined by quantitative RT-PCR. Relative expression of *CaERF14* gene in pepper seedlings after different treatments [NaCl, mannitol, heat, cold and *R. solanacearum*]. Mock represented plant with the mock treated as the control. Error bars represent the mean ± SD of three independent biological replicates. Asterisks indicate statistical significance *0.01 < *P* < 0.05, **P < 0.01, Student’s t-test.

In addition, under the abiotic stresses of drought, high salt, high temperature, and cold treatments (Fig. 4), the expression of *CaERF14* in drought with 400 mM mannitol treatment indicated a significant increase within 1 h of the drought treatment, reaching 4.8-fold of the control through qRT-PCR analyses Subsequently, the expression level decreased gradually. After treatment with 250 mM NaCl, the *CaERF14* gene exhibited up-regulated expression, 1.9-fold higher than that in the control within 1 h, and then down-regulated expression between 3 and 48 h. However, the *CaERF14* expression could not be induced under higher temperature (42 ℃) and low temperature (4 ℃) stress. Moreover, the *CaERF14* expression in biotic stress was up-regulated after inoculation with *R. solanacearum*, gradually increasing from 2 to 96 h. The relative expression level reached the peak at 96 h, which was 2.4-fold of the control. The data showed that it was slightly down regulated at 12 h, which may be due to the fact that there was no significant qRT-PCT performance at 12 h after inoculation with *R. solanacearum* (Fig. 4), or even lower than the background value. However, after 36 h, the gene performance increased significantly. These results indicated that *CaERF14* may be involved in the response to drought, salinity, and *R. solanacearum* inoculation in pepper seedlings. *CaERF14* expression could not be induced by higher temperature and low-temperature stress.

### Analysis of *CaERF14* binding ability to GCC-box and CRT/DRE cis-elements

**Fig. 5:**
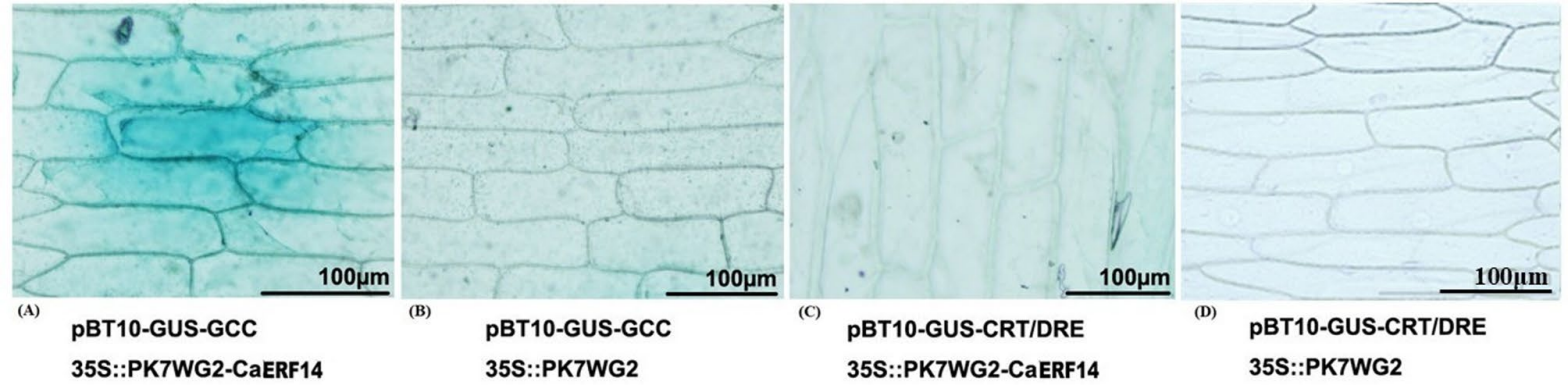
Transient expression analysis of *CaERF14* through bombarding the epidermal cells of onion. **(A)** The expression of GUS gene was detected by X-Gluc staining. **(B-D)** GUS staining was undetectable to the epidermal cells of onion.

The expression of *CaERF14* under different stress conditions suggests that this gene may activate many stress response genes by binding one or two *cis-*acting elements. To test this hypothesis, we constructed a 2X GCC-box vector containing the core sequence pBT10-GUS-2GCC, a 2X *CRT/DRE* vector containing pBT10-GUS-2 CRT/DRE, and a 35 S::PK7 WG2 vector containing the *CaERF14* target gene. Simultaneously, pBT10-GUS-2GCC, pBT10-GUS-2 CRT/DRE, and 35 S::PK7 WG2 vectors were used as the negative controls (Fig. 5B and D). Through bombarding epidermal cells of onion, the transient expression of downstream GUS gene was detected by X-Gluc staining, and the binding ability of *CaERF14* to GCC-box and CRT/DRE *cis*-elements was analyzed. The results showed that when *CaERF14* and pBT10-GUS-2GCC were co-transformed, GUS gene expression could be detected in onion epidermis (Fig. 5A). However, when *CaERF14* co-transformed with pBT10-GUS-2 CRT/DRE, GUS staining could not be detected in onion epidermal cells (Fig. 5C). These results demonstrate that *CaERF14* interacts with GCC-box to regulate the expression of downstream related genes.

### Subcellular localization analysis of *CaERF14*

**Fig. 6:**
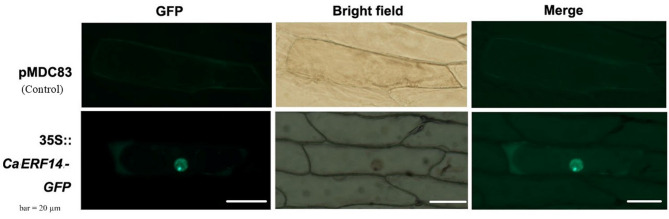
Subcellular localization of *CaERF14*-GFP proteins through bombarding the epidermal cells of onion. pMDC83 is the control group, and Merge is the merged graph that combines GFP and Bright field.

The network resource library WOLF PSORT (http://wolfpsort.org/) was used to predict that the gene is mainly located in the nucleus and primary cell wall, determining the location of the *CaERF14* gene in plant cells. We constructed a 35::*CaERF14*-GFP expression protein, taking the pMDC83 empty vector as the control, and bombarding the onion epidermis with a gene gun to observe the expression of the fusion protein. The results showed that the GFP protein was located in the nucleus and part of the cell membrane (Fig. 6).

### *CaERF14* gene transformed into tobacco

**Fig. 7:**
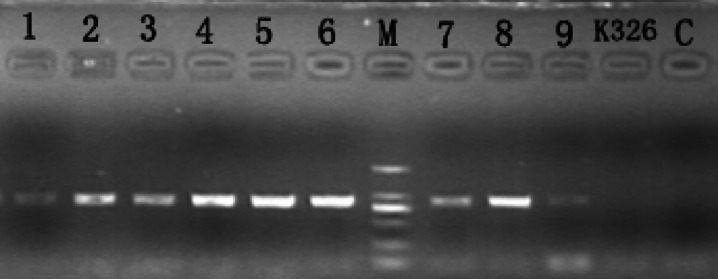
PCR analysis the *CaERF14* gene in T1 tobacco plants of the transgenic lines. PCR amplification was performed with *CaERF14* target gene specific primers (Table 1). The image was modified with the edge of the electrophoresis membrane, showing the bright band of the PCR product. M: DL2000 marker; C : H_2_0 negative control; K326 : wild-type as negative control; 1–9 : transgenic plants.

We obtained nine transgenic tobacco seedlings through *Agrobacterium* mediated transformation. The target gene *CaERF14* was detected and confirmed to have been integrated into the tobacco genome. After screening T1 generation plants through Kan^r^ resistance, the total RNA of T1 generation tobacco leaves was extracted, and PCR detection was performed using gene-specific primers. Compared to the negative control groups (H_2_0 and K326), transgenic tobacco plants could amplify the bands of the target PCR product, whose size and position were consistent with the DL 2000 markers (Fig. 7), indicating that the *CaERF14* gene has been successfully transformed into tobacco.

### Response of Transgenic *CaERF14* tobacco to NaCl stress

**Fig. 8:**
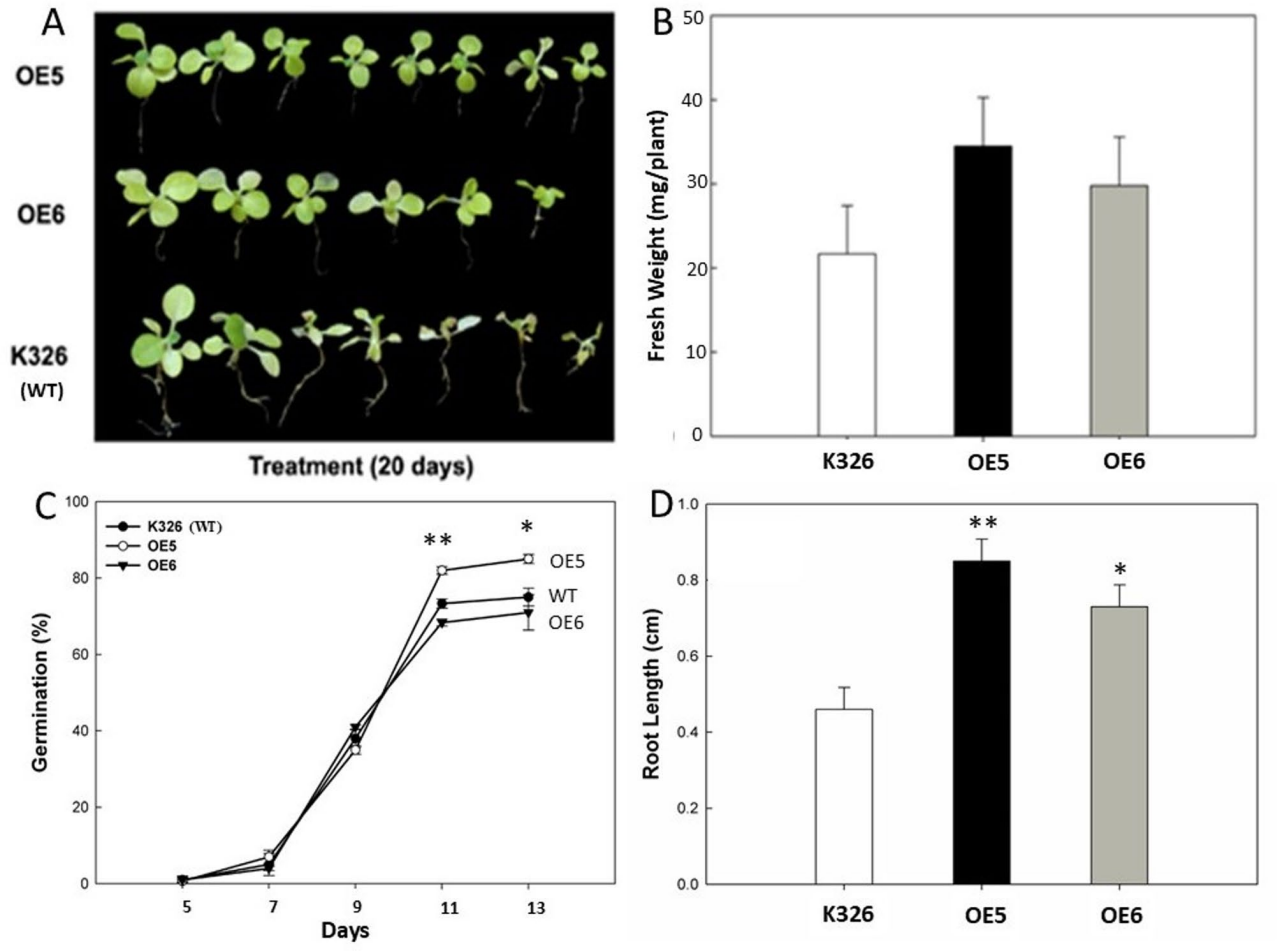
Phenotypes of wild-type and transgenic tobacco lines was treated with NaCl in vitro culture. **(A-B)** Growth of WT and transgenic lines *CaERF14*-OE5 and *CaERF14*-OE6 were inculcated with MS medium containing 300 mM NaCl for 20 days using 2-week-old seedlings. **(C-D)** Seed germination and root length of WT and transgenic plants were inculcated with MS medium containing 150 mM NaCl after 20 days. Data shows mean ± SE of three independent experiments, eight plants for each experiment. Asterisks indicate statistical significance (*0.01 < *P* < 0.05, **P < 0.01, Student’s t-test) between the transgenic and WT plants.

Among the nine selected transgenic lines, we assessed the expression level of *CaERF14* gene in T1 generation plants using quantitative RT-PCR. Two transgenic lines, *CaERF14*-OE5 and *CaERF14*-OE6, were selected for phenotype comparison with wild-type tobacco K326 (WT). Phenotypic changes of WT and transgenic tobacco lines were inducted with MS medium containing 300 mM NaCl for 20 days using 2-week-old seedlings in vitro culture. The results showed that the leaves of the two transgenic lines were greener in color and exhibited less wilting compared to the wild-type K326, while many WT plants displayed wilting, yellowing, and even withering (Fig. 8A). Figure 8B revealed that although the average fresh weight of transgenic tobacco *CaERF14*-OE5 and *CaERF14*-OE6 plants was higher than that of the control WT plants, with approximately 25–40 mg per plant. However, after 20 days of treatment with 300 mM NaCl, there was no significant difference between the transgenic and wild-type tobacco treatment groups (Fig. 8B).

To assess seed germination on salt tolerance, transgenic and WT tobacco seeds were inoculated into MS medium containing 150 mM NaCl. In the control experiment, *CaERF14*-OE5, *CaERF14*-OE6, and K326 seeds were aseptically sown on MS medium only. There was no significant difference in seed germination between the two T1 transgenic lines and WT, indicating that they were all above 95%. However, aseptic seeds were carried out on an MS medium containing 150 mM NaCl, and the germination rate of all three lines tested was significantly reduced to 70–90% (Fig. 8C). Among the salt tolerance of seeds, transgenic tobacco *CaERF14*-OE5 had the highest germination rate, with the statistical significance compared to the wild-type and the *CaERF14*-OE6. In addition, the germination rate of *CaERF14*-OE6 was slightly lower than that of the WT. However, according to the Student’s t-test statistics, there was no significant difference in salt tolerance between *CaERF14*-OE6 and WT seeds (Fig. 8C). Simultaneously, the changes in root length seedlings after germination in response to 150 mM NaCl stress were assessed. Under non-NaCl conditions, there was no significant change in root length for transgenic and WT seedlings. However, after 150 mM NaCl treatment, the root length of transgenic and WT tobacco showed significant changes. After 20 days of germination, the root length of both T1 transgenic seedlings was significantly higher than that of WT, especially the roots of *CaERF14*-OE5, which could grow normally in the NaCl-containing medium (Fig. 8D).

**Fig. 9:**
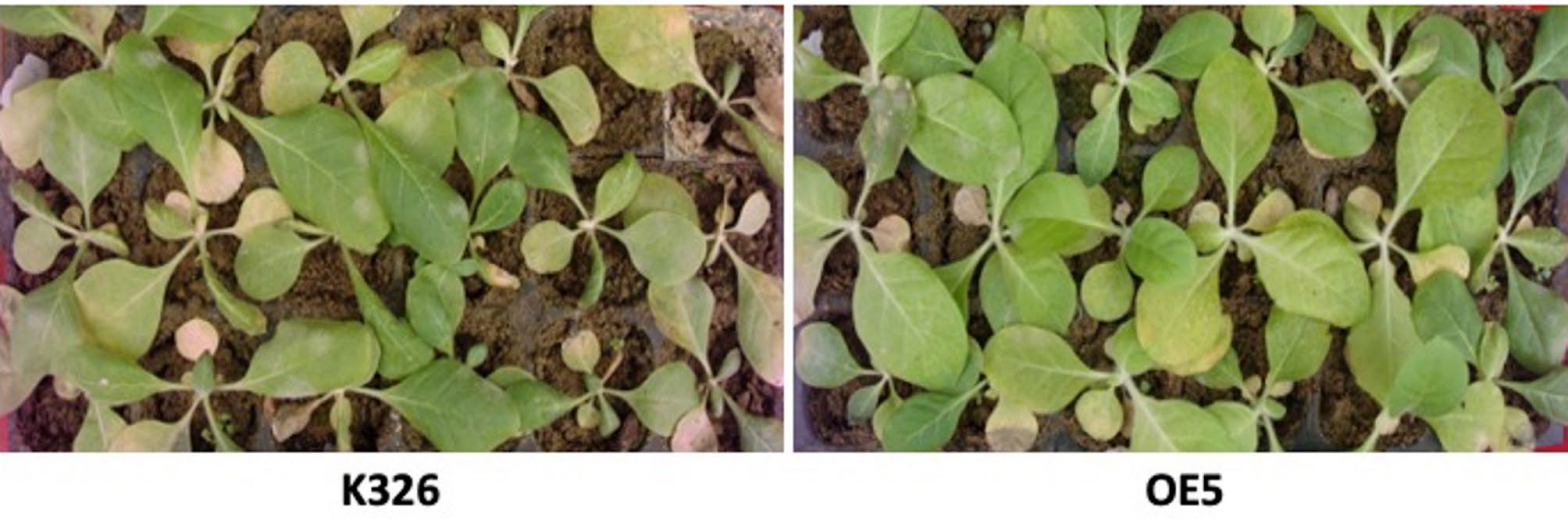
Growth of transgenic (right) and wild-type (left) tobacco seedlings after continuous irrigation with 300 mM NaCl for 15 days using 8-week-old seedlings. Pour 1 L of solution every 40 cells of a plug tray.

**Fig. 10:**
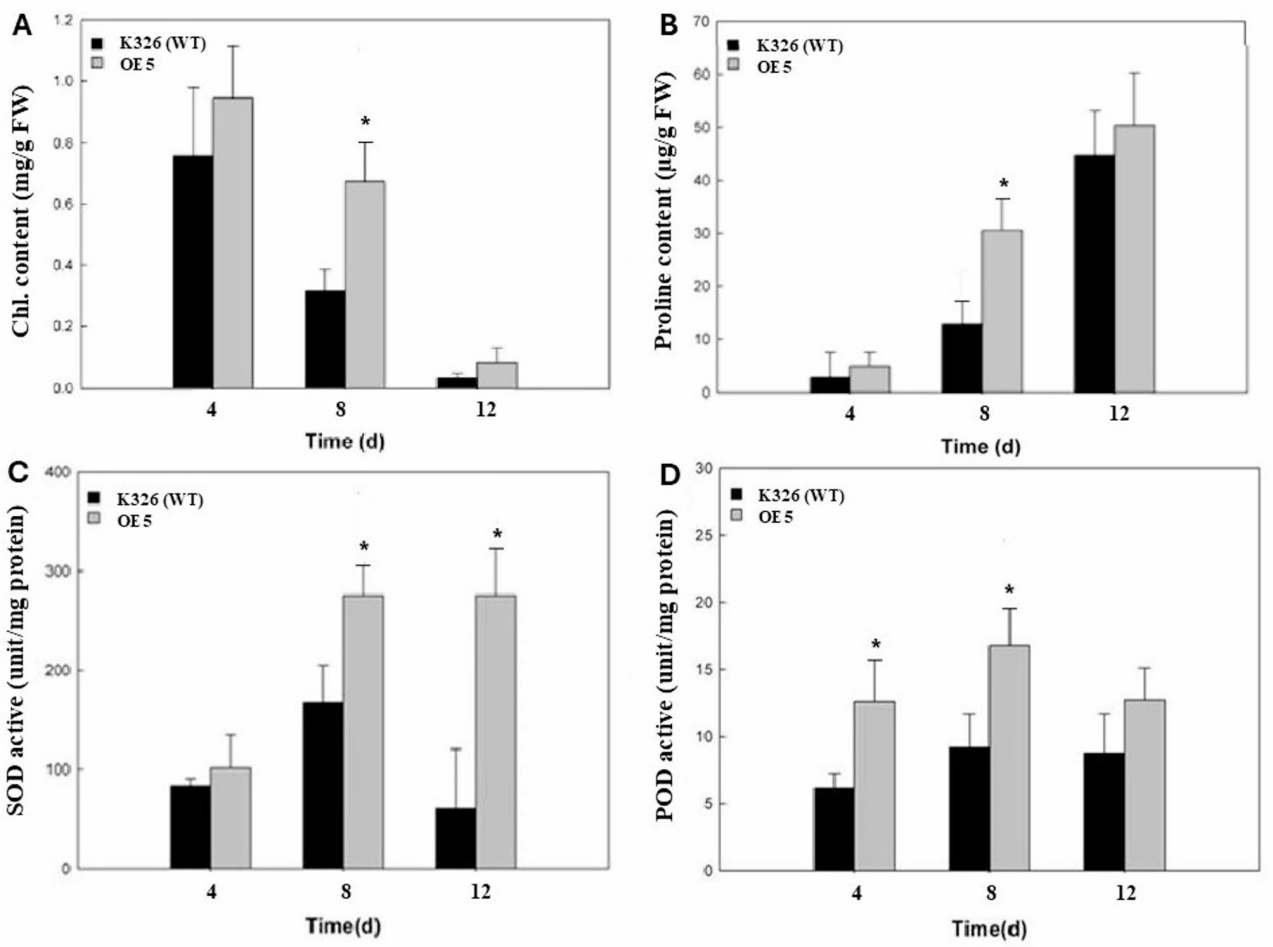
Physiological and biochemical analysis of transgenic tobacco *CaERF14*-OE5 and WT K326 under salt stress. **(A)** The total chlorophyll content, **(B)** free proline content, **(C)** SOD activity, and **(D)** POD activity. Asterisks indicate statistical significance (*P* < 0.05, Student’s t-test) between the transgenic and WT plants.

The transgenic tobacco *CaERF14*-OE5 exhibited superior performance in in vitro culture for tolerance to NaCl. The 8-week-old seedlings grown in the 40 cells of a plug tray were continuously irrigated with 1 L of 300 mM NaCl solution for every plug for 15 days to observe the phenotypic changes of transgenic tobacco and WT seedlings under salt stress. It was observed that the leaves of 73% transgenic tobacco plants were more stretchy and brightly colored, while the WT seedlings showed yellowing or even death (Fig. 9). Physiological and biochemical analysis of transgenic tobacco *CaERF14*-OE5 and WT K326 under this high salt condition. The chlorophyll content decreased gradually between the two, but showed a significant difference on the 8 th day, and both decreased significantly on the 12 th day (Fig. 10A). Free proline content of transgenic tobacco *CaERF14*-OE5 was always higher than that of WT and showed an upward trend with time (Fig. 10B). In addition, the activities of antioxidant enzymes SOD and POD in transgenic *CaERF14*-OE5 plants were significantly higher than those in WT (Fig. 10C, D). Therefore, compared with WT tobacco, transgenic tobacco showed stronger salt tolerance for seed germination or seedling growth in ex vitro or in vivo culture, this gene has a salt tolerance function.

### Response of Transgenic *CaERF14* tobacco to dehydration stress

**Fig. 11:**
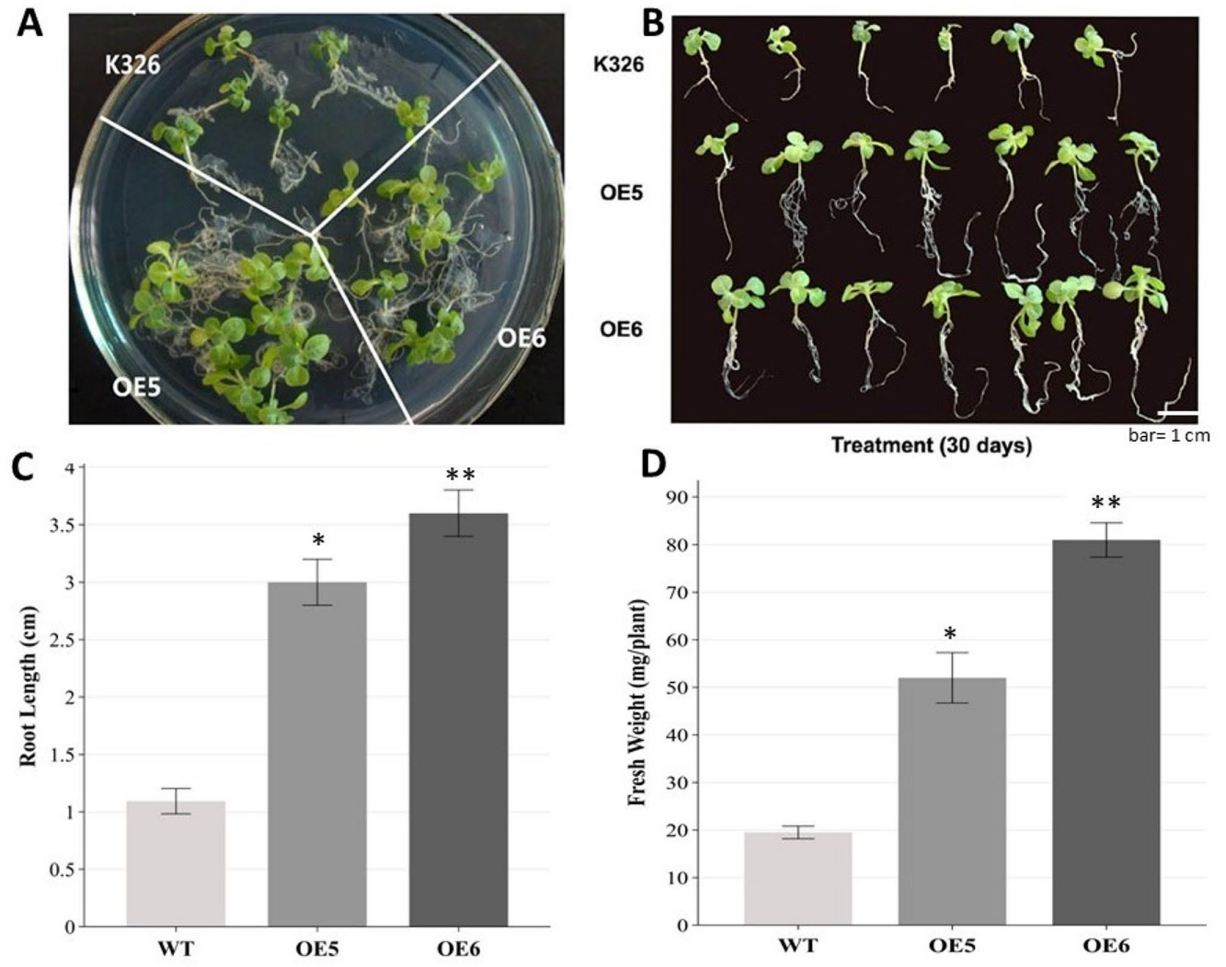
Wild-type and Transgenic line phenotypes under mannitol stress.**(A)** and **(B)** Wild-type K326 and Transgenic line *CaERF14*-OE5、*CaERF14*-OE6 phenotypes under 350 mM mannitol stress. **(C-D)** The root length and fresh weight of Wild-type K326 and Transgenic line *CaERF14*-OE5、*CaERF14*-OE6 under 350 mM mannitol stress for 20 days. Data are mean ± SE of three independent experiments, eight plants for each experiment. Asterisks indicate statistical significance (*0.01 < *P* < 0.05, **P < 0.01, Student’s t-test) between the transgenic and WT plants.

Mannitol was used to simulate the drought stress in plants in an in vitro culture environment. Phenotypic changes of WT and transgenic tobacco lines were induced with MS medium containing 350 mM mannitol for 30 days using 2-week-old seedlings in vitro culture. The results revealed that compared to transgenic tobacco plants, WT tobacco exhibited shrunken and yellowed leaves, significantly shorter plants, shorter roots, and fewer fibrous roots (Fig. 11A, B).

To test seed germination under osmotic stress, transgenic and WT tobacco seeds were inoculated onto MS medium containing 350 mM mannitol. In the control experiment, *CaERF14*-OE5, *CaERF14*-OE6, and K326 seeds were aseptically sown on MS medium only. There was no significant difference in seed germination between the two T1 transgenic lines and WT, indicating that they all exceeded 95%. After mannitol treatment, the germination rate of transgenic tobacco and WT seeds decreased significantly. The germination rate of transgenic tobacco seeds *CaERF14*-OE5 and *CaERF14*-OE6 was higher than those of WT tobacco, at 82%, 79%, and 59%, respectively. At the same time, changes in the root length changes of seedlings after germination in response to 350 mM mannitol were assessed. Under conditions without mannitol, there was no significant change in root length for transgenic and WT seedlings. However, after 350 mM mannitol treatment, the root length of transgenic and WT tobacco exhibited significant changes. After 30 days of germination, the root length of both *CaERF14*-OE5 and *CaERF14*-OE6 transgenic seedlings was significantly higher than that of WT (Fig. 11B), especially, the roots of *CaERF14*-OE6, which could grow normally in a culture medium simulating drought condition (Fig. 11C). The root growth status also reflects the biomass of transgenic tobacco *CaERF14*-OE5 and *CaERF14*-OE6, with significantly higher fresh weight than WT. Especially, *CaERF14*-OE6 has the best growth status under simulated drought in the in vitro environment (Fig. 11D).

**Fig. 12:**
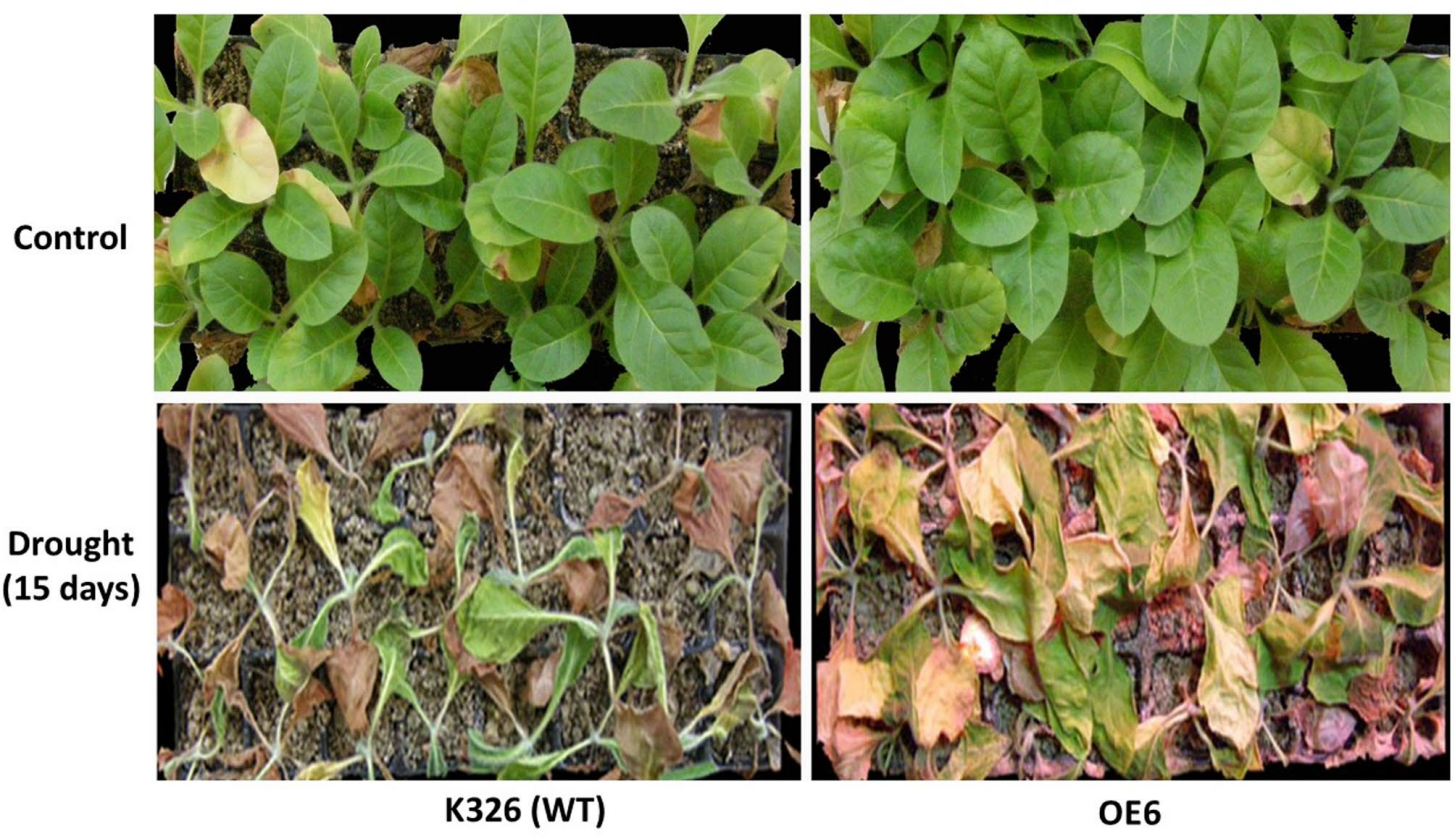
Growth of transgenic *CaERF14*-OE6 (right) and wild-type K326 (left) tobacco seedlings using 8-week-old seedlings. The seedlings were initially grown in pots filled with compost soil under a normal watering regime for 3 weeks. Then, the plants were subjected to a 15-day water cut-off treatment, followed by sufficient watering for 3 days (as shown in the figure below). Well-watered plants were used as the negative control (above photographs).

**Fig. 13:**
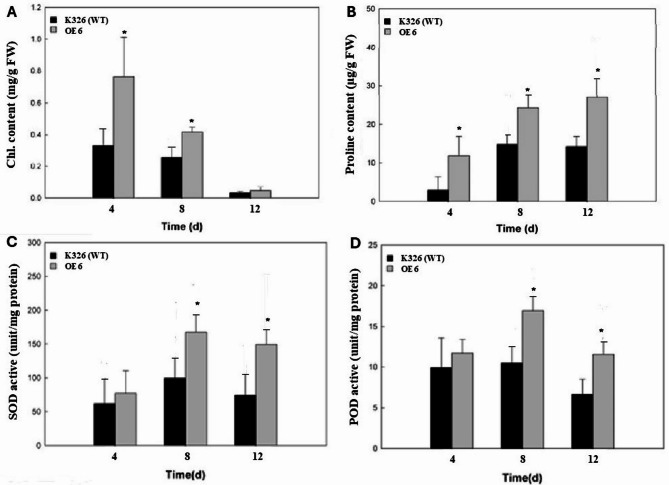
Physiological and biochemical analysis of transgenic tobacco *CaERF14*-OE6 and WT K326 under drought stress. **(A)** The total chlorophyll content, **(B)** free proline content, **(C)** SOD activity, and **(D)** POD activity. Asterisks indicate statistical significance (*P* < 0.05, Student’s t-test) between the transgenic and WT plants.

The transgenic tobacco *CaERF14*-OE6 exhibited superior performance in in vitro culture for tolerance to mannitol. Therefore, the 8-week-old T1 transgenic and wild-type tobacco seedlings were initially grown in pots filled with compost soil under a normal watering regime for 3 weeks. Irrigation was then withheld from the soil-grown plants for 15 days, followed by re-watering. Survival rates were observed 3 days after re-watering. After 15 days of water deprivation, the soil exhibited a very dry state. In response to soil drought stress of transgenic tobacco *CaERF14*-OE6 and WT seedlings, after 15 days, both plants exhibited a state of drought and dehydration, with seedlings turning yellow or even dying (Fig. 12, below). The control group of *CaERF14*-OE6 and WT seedlings was only irrigated with water and still grew normally (Fig. 12, above). Although the seedlings showed less drought resistance, it could still be seen that the growth of *CaERF14*-OE6 transgenic plants was stronger than that of WT (Fig. 12). Physiological and biochemical analysis of transgenic tobacco *CaERF14*-OE6 and WT K326 under the drought conditions, the chlorophyll contents of transgenic tobacco *CaERF14*-OE6 were significantly higher than that of WT K326 on the 4 th and 8 th days, and both decreased on the 12 th day (Fig. 13A). The free proline content of transgenic tobacco *CaERF14*-OE6 was always higher than that of WT and showed an upward trend with time (Fig. 13B). The activities of antioxidant enzymes SOD and POD in transgenic *CaERF14*-OE6 plants were significantly higher than those in WT (Fig. 13C, D). Based on the above results, the *CaERF14* gene derived from pepper was transformed into tobacco. Regardless of the mannitol tests for seed germination or seedling growth in in vitro culture or in soil drought stress, this gene has a drought tolerance function.

## Discussion

Gene structure analysis plays a crucial role in revealing gene function. This study isolated and obtained a new ERF in pepper, named *CaERF14*, belonging to the subfamily Xa (B-4) of the ERF family (Fig. 1). Sequence analysis indicates that the gene contains a conserved AP2/ERF domain, with an N-terminus rich in serine, which is a characteristic of transcription factors. The conservation of alanine and aspartate at the 14 th and 19 th positions of the AP2/ERF domains^40^. Multiple sequence alignment showed the conservation of the AP2/ERF domain in ERFs from different plants, and there are few reports about this subfamily gene. Existing reports suggest that this gene type could respond to exogenous hormones and abiotic stress^26,27,35^.

Relative expression of *CaERF14* in response to various biotic and abiotic treatments was determined by qRT-PCR, including exogenous hormones and abiotic stress (Figs. 3 and 4). The phytohormone abscisic acid (ABA) is known to regulate processes that protect plants from damage induced by drought. The ABA-responsive transcription factors containing a bZIP domain bind to the ABA-responsive element in the promoter of downstream genes^7,41,42^. *ERF2* plays a key role in salicylic acid (SA) and jasmonic acid (JA) signaling pathways to confer resistance to invasion by *S. lycopersici* and chilling response^39,38^. The *CaERF14* is a biochemical reaction induced by drought, salt, and *R. solanacearum* (Fig. 4). Overexpression of ERF genes could enhance resistance to *Pseudomonas* invasion by regulating the expression of GCC box-containing genes^38^. Previous studies have identified ERF family members in pepper; the genes could not only respond to the infection of pathogenic bacteria but also exhibit stress tolerance to cold, high salt levels, and drought^8,35^. In addition, transcription factors (TFs) play essential roles in regulating the expression of specific resistance-related genes in various defense response pathways. ERF TFs were shown to regulate the expression of PR genes by binding to GCC (AGCCGCC) box-containing genes in their promoter regions^43^. The results indicate that the *CaERF14* gene participates in certain plant biological and abiotic stress response functions.

Numerous transcription factors are known to play key roles in the response of plants to various stresses, regulating the upregulation of downstream genes by binding to *cis*-acting elements in the promoter region of stress-related genes^41^. Subcellular localization experiments showed that the fusion protein of 35 S::*CaERF14*-GFP was also located in the nucleus (Fig. 6), while transient expression experiments demonstrated that the gene can bind to the GCC-box *cis*-element, but not to the DRE/CRT element (Fig. 5). ERF genes such as *Pti4/5/6*, *MACD1*, *GmERF5*, and *StERF3* are in the nucleus and play important and distinct roles in the stress tolerance of plants^44–47^. The DREBA subfamily can recognize the drought-induced element DRE (TACCGACAT) and the low-temperature-induced element CRT (AGCCGAC)^48^, participating in the ethylene signaling pathway to help plants resist the effects of stress^13,48,49^.

In addition, the analysis of gene expression sites in plant tissues can preliminarily predict the future application of this gene in environmental stress. For example, compare the ERF group between *CaERF14* and *CaPTI1* of peppers. The tissue-specific expression of the *CaERF14* gene shows a high qRT-PCR level in the flower and stem parts, while this gene exhibits very low expression in the roots (Fig. 2). However, the expression of the *CaPTI1* gene was highest in the roots, almost 26 times that of the leaves^35^. The result shows that the genes of ERF group in pepper are not stabilized in specific plant tissues, and the *CaERF14* gene is strongly expressed in aboveground parts, so it may predict better environmental drought resistance and salt stress tolerance, while the *CaPTI1* gene is strongly expressed in underground parts, so it can predict better resistance to soil pathogens^26,50^.

Several extensive studies have been conducted on many ERF family proteins in plant species through overexpression and transgenics. However, studies on ERF family proteins with negative regulatory functions are very few^51^. We cloned the *CaERF*14 gene derived from pepper and transformed it into tobacco, observing its functional expression. The response of transgenic *CaERF14* tobacco both in ex vitro and in vitro culture environments showed that the gene has functions related to salt resistance and dehydration tolerance (Figs. 8, 9, 10, 11, 12 and 13). High salt stress promotes ethylene biosynthesis, which can activate the downstream network and result in altered gene expression^52^. Through research on transgenic and mutant strains, the sequence of ethylene response under salinity stress was elucidated. These studies confirm that ethylene has a positive or negative regulatory effect on salt resistance^53,54^. Regardless of the mannitol tests for seed germination or seedling growth in vitro culture or in soil drought stress, the *CaERF14* gene exhibits drought tolerance function. Transgenic *CaERF14* tobacco plants show heightened abilities in drought and salt resistance abilities, indicating that the *CaERF14* gene can accelerate the activation of the antioxidant defense system in plants. Transgenic lines *CaERF14*-OE5 and *CaERF14*-OE6 contained higher levels of antioxidant proline compared to wild-type plants, and their activities of SOD and POD enzyme were higher than wild-type plants (Figs. 10 and 13). This antioxidant capacity is not only achieved by activating the expression of osmotic related genes, but also by triggering the expression of ROS oxidative related genes^55^.

The gene function expression provided by *A. tumefaciens* gene transfer system in tobacco is the most stable and feasible, which could obtain the characteristics of salt and drought tolerance of *CaERF14*. Although the physiological characteristics of salt and drought resistance could be expressed in transgenic tobacco. But the next step is to verify the overexpression of *CaERF14* gene in transgenic Pepper. The advanced breakthrough genome engineering tool CRISPR/Cas9 and virus-induced gene silencing (VIGS) technology have been effectively utilized in editing specific fields in ERFs^51,56^. By the *CaERF14* gene transfer system, the salt tolerance and drought tolerance characteristics of transgenic crops (such as pepper) can be enhanced. Crops with stronger stress resistance can improve crop yield and resilience in adversity. The development of highly durable transgenic crops in accurate genome editing can meet the growing demand for flexible agricultural products in emerging markets.

## Methods

### Plant materials and growth conditions

Pepper (*C. annuum* L.) from local species 8# of Fujian China and tobacco cultivar K326 plants were utilized in this study. Seeds were sown in a steam-sterilized compost soil mix (peat moss, perlite, and vermiculite, 5:3:2, v/v/v), sand, and loam soil (1:1:1, v/v/v). The pepper and tobacco plants were cultivated in a growth chamber at 26 ± 1 °C under white fluorescent light (130 µmol photons·m^−2^·s^−1^, 16 h of light and 8 h of darkness per day).

Different exogenous hormones (abscisic acid (ABA), methyl jasmonate (MeJA), Ethephon (ETH), salicylic acid (SA) and auxins) were sprayed on the leaf surfaces, and abiotic stress (high temperature, low temperature, drought, and high salt) were applied to treat pepper seedlings with similar growth status for 25 days^35^. Additionally, 8-week-old pepper seedlings were subjected to bacterial wilt (*R. solanacearum*) solution. The samples were collected at 0, 1, 3, 6, 12, 24, 48, 72 and 96 h respectively^38^. The total RNA of plants was extracted using the Trizol method (Invitrogen, Thermo Fisher Scientific, China). Each treatment was replicated three times.

In vitro culture of tobacco involved soaking seeds of tobacco wild-type K326 and the T1 transgenic lines in this study were soaked in 75% alcohol for 20 s, rinsing in sterile water three times, and then soaking in 10% H_2_O_2_ for 8 min. Finally, the seeds were washed in sterile water three to five times and transferred to MS medium for germination.

### Cloning and sequence analysis of *CaERF14*

The positive clones of the pepper cDNA library were randomly selected and sequenced with M13 reverse (NovoPro Company, Shanghai, China) as the sequencing primer and obtained a DNA sequence. Comparison of the domain with NCBI (http://www.ncbi.nlm.gov/) and DNAMAN 9.0 version software (Lynnon Biosoft, USA), the gene sequence and open reading frame were used to identify putative the AP2/ERF domain-containing genes through BLAST (http://blast.ncbi.nlm.nih.gov/Blast.cgi). Specific primers were designed to obtain positive clones of the target genes by PCR from the cDNA library. The PROSITE database (https://prosite.expasy.org), SMART (http://smart.embl-heidelberg.de/) and WOLF PSORT (http://wolfpsort.org/) were used to predict and screen the physical and chemical properties of the protein amino acid sequences of all ERF transcription factors, such as protein length and molecular weight. ClustalX 2.0 version software was used to perform multisequence alignment of the conserved AP2 domains in the ERF family of pepper and *A. thaliana*, and the results were imported into MEGA 4.1 version software to construct the rootless evolutionary tree of the ERF family.

### Vector construction

The pepper expression vector was constructed using Gateway vector construction technology (Invitrogen, Thermo Fisher Scientific, China). Plasmid pDONR207 was served as the entry vector, with the overexpression target vector being plasmid pK7 WG2 containing the CaMV35S promoter. The subcellular localization target vector was plasmid pMDC83, incorporating the CaMV35S promoter and the green fluorescent protein gene (GFP). The positive clone plasmid obtained through screening was used as a template, and the designed overexpression primers and subcellular localization primers were employed, respectively (Table 1).

PrimeSTAR™ HS DNA polymerase (Takara Biomedical Technology (Beijing) Co., Ltd.) was used for amplification, with the attB linker was added on both sides of the target gene (Table 1). The PCR protocol was as follows: a 25 µL system, pre denatured at 95 °C for 2 min, denatured at 94 °C for 15 s, annealed at 55 °C for 30 s, extended at 68 °C for 60 s/90 s, and 10 cycles constituted the first round of amplification. In the second round of amplification, the 10 µL of the first round of amplification was used as the template, and the primer was the linker primer attB1/attB2 (Table 1). The amplification procedure was carried out in a 50 µL system, involving pre-denaturation at 95 °C for 1 min, denaturation at 94 °C for 15 s, annealing at 45 °C for 30 s, extension at 68 °C for 60 s/90 s, and the number of amplification cycles was 5; Denaturation at 94 °C for 15 s, annealing at 55 °C for 30 s, extension at 68 °C for 60 s/90 s, 20 cycles of amplification, and extension at 68 °C for 5 min. The product of attB-PCR gel was recovered for BP reaction and the target gene was cloned into pDONR207 entry vector. The 8 µL reaction system included 15–150 ng of attB-PCR product, 150 ng of pDONR207 plasmid, 2 µL of BP Clonase™ enzyme mix. Subsequently, a warm bath was maintained at 25 ℃ for 1 h. After overnight, 1 µL proteinase K was added and digested at 37 ℃ for 10 min to transform into *E. coli* DH10B competent cells (Kalang Biotech. Co., Shanghai China).

The target genes were cloned into the target vectors pK7 WG2, PYL279 and pMDC83. The 10 µL reaction system comprised 50–150 ng of primer clone, 150 ng of target vector plasmid, and 2 µL of LR Clonase™ enzyme mix. After a warm bath at 25 ℃ for 1 h, 1 µL proteinase K was added and digested at 37 ℃ for 10 min. Subsequently, the reaction product was transformed into *E. coli* DH10B. The obtained plasmid served as a template for PCR validation.

The vectors for transient expression analysis were commissioned by Shanghai Biological Company to synthesize GCC-box and CRT/DRE elements with sticky ends at both ends of the SpeI and XbaI sites of the restriction enzymes. The cis-acting elements of GCC-box and CRT/DRE were recombined with the pBT10-GUS vector, transformed into the *E. coli* DH10B, and the obtained plasmid was used as a template for PCR validation. After sequencing, they were named pBT10-GUS-GCC and pBT10-GUS-CRT/DRE for transient expression analysis.

**Table 1:** Primers of over-expression, subcellular localization andreporter vector.

Primers	Primer sequences (5’ → 3’)
OE-*CaERF14*-F	5’- AAAAAGCAGGCTTCATGGAAAGAAGGTCGAAAGA − 3’
OE-*CaERF14*-R	5’ - AGAAAGCTGGGTCTTACTTTCCGTGTTCAAACT − 3’
GFP-*CaERF14*-F	5’- AAAAAGCAGGCTTCAATGGAAAGAAGGTCGAA − 3’
GFP-*CaERF14*-R	5’- AGAAAGCTGGGTCCTTTCCGTGTTCAAACTCTT − 3’ (Remove stop codon)
attB1	5’ - GGGGACAAGTTTGTACAAAAAAGCAGGCT − 3 ´
attB2	5’- GGGGACCACTTTGTACAAGAAAGCTGGGT − 3’
GCC-F	5’- CTAGTC*AGCCGCC*AAAGAGGACCCAGAATC*AGCCGCC*AAAGAGGA CCCAGAATT − 3’
GCC-R	5’- CTAGAATTCTGGGTCCTCTTT*GGCGGCT*GATTCTGGGTCCTCTTT *GGCGGCT*GA − 3 ´
DRE/CRT-F	5’- CTAGTTAAATAAAAGATATACT*ACCGAC*ATGAGTTCCAAAAGCTAAAT AAAAGATATACT*ACCGAC*ATGAGTTCCAAAAGCT- 3’
DRE/CRT-R	5’- CTAGAGCTTTTGGAACTCAT*GTCGGT*AGTATATCTTTTATTTAGCTTTT GGAACTCAT*GTCGGT*AGTATATCTTTTATTTAA − 3’

The underline letters represent the addition of SpeI and XbaI restriction sites, while the italic letters represent the core sequences of GCC-box and DRE/CRT.

### *CaERF14* gene expression profile analysis

Transient expression analysis was conducted using the Biolistic PDS-1000/He (BioRad) particle bombardment method. The inner epidermis of onion was removed and then laid on MS medium and inoculated at room temperature in darkness for 24 h. The inner epidermis were bombarded with 5 µg pK7 WG2 plasmid containing the target gene, 5 µg pBT10-GUS-GCC and 5 µg pBT10-GUS-CRT/DRE plasmids, respectively. In addition, only 5 µg pBT10-GUS-GCC and 5 µg pBT10-GUS-CRT/DRE plasmids were used as negative controls, respectively. After bombardment, the onion epidermis was laid on MS medium (2% agar, 3% sucrose, pH 5.8) and incubated at dark room temperature for 24 h. X-Gluc staining was performed to observe the cells of onion epidermis, and photos were taken under a microscope.

Subcellular localization analysis was carried out by bombarding the inner epidermal cells of onions (the same method as above). The coding region of the *CaERF14* gene without the stop codon was inserted into a binary vector, pMDC83-GFP, which contains a cauliflower mosaic virus (CaMV) 35 S promoter and a green fluorescent protein (GFP) tag at the carboxyl end of the insert. The pMDC83 empty vector was the negative control. The cells were observed under an Olympus fluorescence microscope, and images were collected from the same field of view of white light and blue excitation.

### RNA isolation and quantitative RT-PCR analysis of gene expression

Total RNA was extracted from the collected sample after various stress treatments using Trizol (Invitrogen, http://www.invitrogen.com/) method. Then the first strand cDNA was synthesized using of prime script TM kit (Takara) following the manufacturer’s protocols.

The iQ^L^ SYBR Green Supermix (Bio-Rad, http://www.bio-rad.com) was specifically formulated for real-time PCR. Subsequently, qRT-PCR was conducted using SYBR Premix Ex TaqTM II (TaKaRa) in an iCycler iQTM Multicolor PCR Detection System (Bio-Rad, USA). The total volume of PCR amplification was 20 µL, with 0.2 µM of forward and reverse primers, 5–10 ng of cDNA template, and the calibration was performed with the addition of ROX Reference Dye II (50X). The amplification cycling parameters of qRT-PCR were as follows: 95 ℃ for 1 min and followed by 40 cycles at 95 ℃ for 10 s, 56 ℃ for 15 s, and 72 ℃ for 15 s. Formula for calculating relative expression quantity: (1) Fold change = 2^−△△Ct^; (2) △△Ct = (CT, gene- CT, action) treatment group - (CT, gene - CT, action) control group^57–60^, used for fluorescence quantitative PCR analysis of primers, R-*CaERF14*-F (5’ – GGGAGTTCGGGAAGAAGGAAGT – 3’), and R-*CaERF14*-R (5’ – TGATGAGGTGGCTGCTGGAA – 3’). The actin gene of pepper was used as an internal reference gene, PCR analysis of primers was actin-F (5’ – AGGGATGGGTCAAAAGGATGC – 3’), and actin-R (5’ – GAGACAACACCGCCTGAATAGC – 3’).

In the abiotic stress and hormone treatments of pepper under indoor conditions (25 ℃, 60–70% relative humidity, 16 h light/8 h dark period), when the seedlings were approximately 25 days old, having grown two true leaves with consistent growth vigor, they were transferred into 40 cells of a plug tray as the healthy pepper seedlings for treatments. The 8-week-old pepper seedlings simulating salt stress conditions were irrigated with 1 L of 250 mM NaCl in each 40-cells plug. Similarly, simulating drought stress conditions were irrigated with 1 L of 400 mM mannitol solution in a plug.

In addition, in the treatments of plant growth regulators, abscisic acid (ABA): 0.1 mM ABA was sprayed on the leaves of peppers in each plug, and H_2_O was used as the control; salicylic acid (SA): 5 mM SA was sprayed on the leaves in each plug, and 10% ethanol as the control; methyl jasmonic acid (MeJA): 0.1 mM MeJA was sprayed, and 10% ethanol as the control; ethylene: 10 mM Ethephon (ET) was sprayed, and H_2_O was used as the control. The solutions for plant growth regulators were sprayed on 8-week-old pepper seedlings of peppers at a volume of 1 L per plug tray. For the treatment of *R. solanacearum*, the pepper seedlings were inoculated with *Ralstonia* (1 × 10^8^ cfu mL^−1^) by root drenching. The leaves of peppers treated with biotic and abiotic stress and the corresponding control plants were collected at different time periods (0–96 h), with three replicates per time. Liquid nitrogen was immediately used to freeze and extract RNAs for subsequent analysis.

### Plant transformation

All the above the target genes mentioned above were cloned into target vectors pK7 WG2, and the constructs were introduced into *A. tumefaciens* (strain EHA105). Tobacco K326 transformation was carried out using the leaf disc method. For each construct, transgenic seedlings were selected, inoculated on MS medium including 1.5 mg L^−1^ BA, 0.1 mg L^−1^ IAA and supplemented with 100 mg/L kanamycin. The presence of the target gene *CaERF14* was detected and confirmed to have been integrated into the tobacco genome. After screening T1 generation plants through Kan^r^ resistance, the total RNA of T1 generation tobacco leaves was extracted with Invitrogen TRIzol™ reagent (ThermoFisher, China), and the cDNA was synthesized by reverse transcriptase M-MLV (RNase H-) (Takara Bio, China). PCR amplification was carried out with *CaERF14* target gene specific primers (Table 1). The target gene fragments were detected by electrophotography, and the transgenic lines were screened.

### The response of the Transgenic plants to salinity and dehydration stress

Tobacco K326 (WT) and T1 transgenic seeds were harvested simultaneously and stored at 4 ℃ for 3 months^61^. For seed germination analyses, approximately 50 seeds were surface-sterilized and sown on MS medium containing 150 mM NaCl, or 350 mM mannito^8,41^. Seeds were vernalized at 4 ℃ for 3 days before growth at 21 ℃ under 16 h light/8 h dark conditions. Seedlings with fully emerged radicle tips and green cotyledons were scored for seed germination. Next, the 8-week-old seedlings grown in the 40 cells of a plug tray with compost soil were continuously irrigated with 1 L of 300 mM NaCl solution each plug, for 15 days to observe the phenotypic changes of transgenic tobacco and WT seedlings under salt stress.

For the drought treatment, the 8-week-old T1 transgenic and wild-type tobacco seedlings were initially grown in pots filled with compost soil under a normal watering regime for 3 weeks. Irrigation was then withheld from the soil-grown plants for 15 days, followed by re-watering. Survival rates were scored 3 days after re-watering. Well-watered plants were used as the negative control.

### Determination of chlorophyll, free proline and antioxidant enzyme activities

Stress treatment of transgenic tobacco and wild-type tobacco, chlorophyll content determination modified by Wintermans and Mots (1965) method^62^. Extraction of chlorophyll from tobacco leaves with acetone: ethanol: water ratio of 4.5 : 4.5 : 1.0 in the dark for 30 min, centrifuged at 4 ℃ for 15 min at 10,000 x g, and the absorption was measured at A_663nm_ and A_645nm_ with a spectrophotometer (Multiskan SkyHigh A51119700 C, ThermoFisher). The chlorophyll concentration was calculated as 20.29 A_645_ + 8.04 A_663_.

For the determination of free proline content, referred to Shan et al. (2007)^63^, fresh leaves (0.5 g) was homogenized and extracted with 5 ml of 3% sulfosalicylic acid and was added and boiled in boiling water for 10 min. Proline content was analyzed by the Ninhydrin method, taking 2 mL of the extraction solution added 2 mL of glacial acetic acid and 3 mL of acid ninhydrin solution, heating in boiling water for 40 min. The reaction mixture was extracted with 3 mL toluene, and the absorbance was measured at 520 nm with an Ultraviolet-visible spectrophotometer. The proline content was determined by a standard curve (Sigma, St Louis, MO, USA).

Superoxide dismutase (SOD) and peroxidase (POD) activities were determined by the methods of 0.3 g fresh leave sampled after liquid nitrogen treatment as a treatment, repeated three times. 50 mmol/L phosphoric acid buffer (pH 7.8, containing 0.2 mol/L EDTA and 2% PVP) 3 ml was added and homogenized^64,65^. The homogenate was centrifuged at 12,000 r/min and 4 ℃ for 20 min. The supernatant was the extract, which was used to determine the activities of the enzymes. SOD activity was measured according to the photochemical reduction of nitrotetrazolium chloride (NBT) method^64^. The activity of POD was determined in absorbance was recorded at 530 nm with a spectrophotometer^65^.

### Statistical analysis

All data were presented as mean ± standard deviation (SD) of three independent replicates. Paired t-tests were performed to assess significant differences using the SPSS Statistics 17.0 software (IBM China Company Ltd., Beijing, China). All experiments were independently repeated 3 times as independent analyses, and each analysis was performed with three biological replicates.

## Electronic supplementary material

Below is the link to the electronic supplementary material.

Supplementary Material 1

## Data Availability

All data generated or analyzed during this study are included in this published article and its supplementary information file.
