# Supplementary material for: Isolation of the AP2/ERF transcription factor CaERF14 in pepper and functional characterization under salinity and dehydration stress
Source: Sci Rep. 2025 Jun 5;15:19726. doi: 10.1038/s41598-025-03808-9 (PMC12141531; doi:10.1038/s41598-025-03808-9)
Supplement: Supplementary file 1 — Supplementary Material 1 [file 41598_2025_3808_MOESM1_ESM.docx]

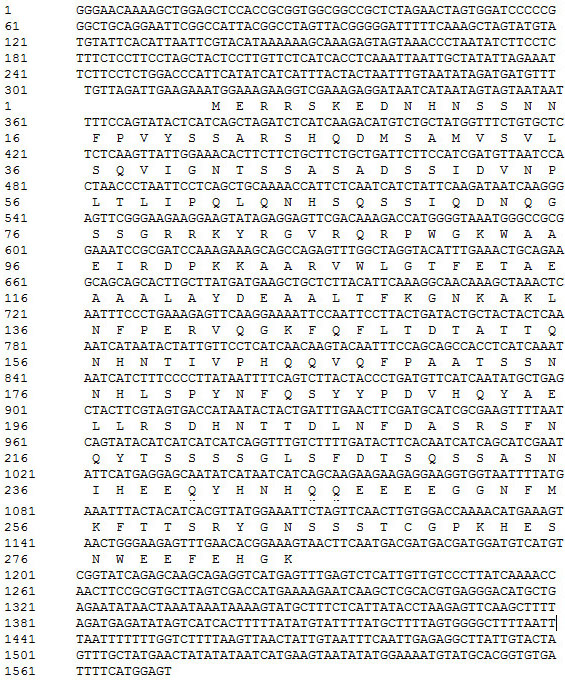


Supplementary Figure S1. The cDNA sequence and deduced amino acid sequence of the *CaERF14* gene.


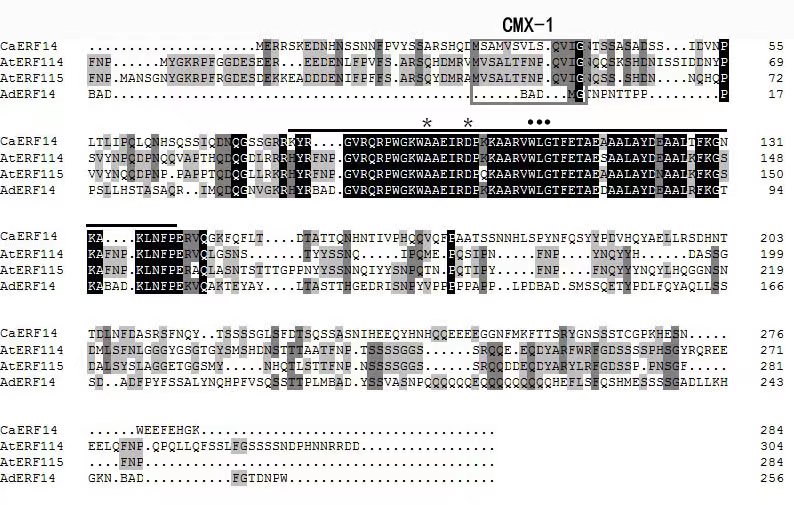


Supplementary Figure S2. Multiple sequence alignment analysis of AP2/ERF domain-containing transcription factor of *CaERF14* and its homologous proteins from other plant species. The number on the right shows the sequence position of the amino acid residue. The overline represents the conserved DNA-binding domain (AP2/ERF domain). The asterisks indicate A14 and D19 ERF specific amino acid residue. The black dots represent the conserved WLG motif. The gray boxed sequence represents the Ⅹa subgroup specific motif. *AtERF114* and *AtERF115* are the AP2 transcription factors of *Arabidopsis*. *AdERF14* is the AP2 transcription factor of kiwifruit.


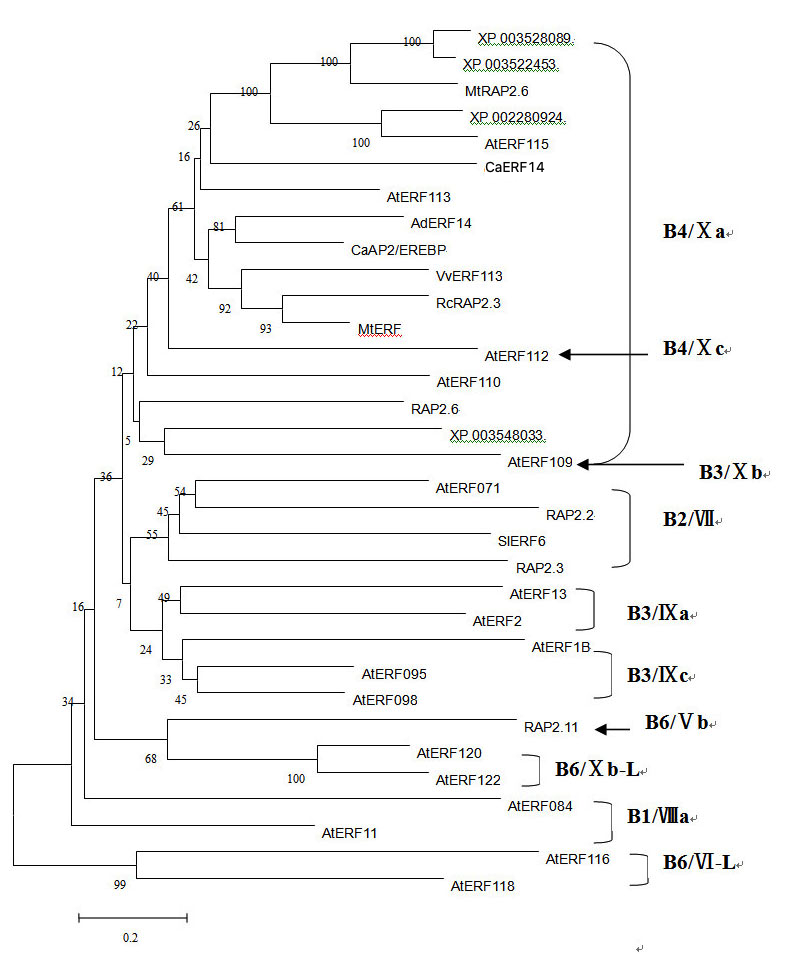


Supplementary Figure S3. Phylogenetic tree shows the relationships of *CaERF14* with other species.

The accession number indicate unnamed genes.
